# Maternal immunization against myostatin suppresses post-hatch chicken growth

**DOI:** 10.1371/journal.pone.0275753

**Published:** 2022-10-06

**Authors:** Rajeev Mishra, Rajesh Jha, Birendra Mishra, Yong Soo Kim

**Affiliations:** Department of Human Nutrition, Food and Animal Sciences, University of Hawaii at Manoa, Honolulu, Hawaii, United States of America; University of Life Sciences in Lublin, POLAND

## Abstract

Myostatin (MSTN) is a negative regulator of skeletal muscle growth, thus it was hypothesized that immunization of hens against MSTN would enhance post-hatch growth and muscle mass via suppression of MSTN activity by anti-MSTN IgY in fertilized eggs. This study investigated the effects of immunization of hens against chicken MSTN (chMSTN) or a MSTN fragment (Myo2) on the growth and muscle mass of offspring. In Experiment 1, hens mixed with roosters were divided into two groups and hens in the Control and chMSTN groups were immunized with 0 and 0.5 mg of chMSTN, respectively. In Experiment 2, hens in the chMSTN group were divided into chMSTN and Myo2 groups while the Control group remained the same. The Control and chMSTN groups were immunized in the same way as Experiment 1. The Myo2 group was immunized against MSTN peptide fragment (Myo2) conjugated to KLH. Eggs collected from each group were incubated, and chicks were reared to examine growth and carcass parameters. ELISA showed the production of IgYs against chMSTN and Myo2 and the presence of these antibodies in egg yolk. IgY from the chMSTN and Myo2 groups showed binding affinity to chMSTN, Myo2, and commercial MSTN in Western blot analysis but did not show MSTN-inhibitory capacity in a reporter gene assay. In Experiment 1, no difference was observed in the body weight and carcass parameters of offspring between the Control and chMSTN groups. In Experiment 2, the body weight of chicks from the Myo2 group was significantly lower than that of the Control or chMSTN groups. The dressing percentage and breast muscle mass of the chMSTN and Myo2 groups were significantly lower than those of the Control group, and the breast muscle mass of Myo2 was significantly lower than that of the chMSTN. In summary, in contrast to our hypothesis, maternal immunization of hens did not increase but decreased the body weight and muscle mass of offspring.

## Introduction

Myostatin (MSTN), a member of the transforming growth factor-β family proteins, is expressed in skeletal muscle and targeted deletion of the MSTN gene in mice increased the muscle mass by 2–3 times [1]. Systemic administration of MSTN [2] or overexpression of MSTN by transgenesis or ectopic gene transfer [3, 4] reduced muscle mass, indicating that MSTN is a negative regulator of skeletal muscle development and growth. Subsequent studies have shown that MSTN inhibition using various approaches including the administration of MSTN-blocking proteins, peptides, or anti-MSTN antibodies [5–10] and delivery of MSTN-blocking genes [11–14] enhanced skeletal muscle growth in laboratory animals. These findings suggested that suppressing the biological activity of MSTN either during embryonic development or the postnatal growth period would be a strategy to enhance the skeletal muscle growth of farm animals or to treat muscle-wasting conditions in humans.

Improving animal growth performance and production efficiency is essential to supply animal proteins to growing human population with minimal environmental footprints. Enhancing skeletal muscle growth improves the efficiency of feed utilization, resulting in improved efficiency of meat-animal production [15–17]. Immuno-neutralization of MSTN has been used in a few studies to improve growth performance in farm animals. It was reported that active immunization of pigs against MSTN significantly increased carcass lean percentage with a decrease in intramuscular fat percentage [18]. In broiler chicken, *in-ovo* administration of monoclonal anti-MSTN antibody increased the body, carcass, leg, and breast muscle weights [9]. The results illustrate the potential of *in-ovo* suppression of MSTN to enhance the growth performance and muscle production in chickens. To avoid the administration of anti-MSTN antibodies to individual animals, some studies investigated the effect of maternal immunization against MSTN on the growth performance of offspring [19–21]. While the studies showed the presence of anti-MSTN antibodies in offspring after maternal immunization against MSTN or MSTN fragments, the results of maternal immunization against MSTN on the growth performance of offspring were not consistent. Improved growth rate and muscle mass of offspring were observed in mice after maternal immunization against MSTN fragments [20, 21], but no effect was observed in offspring from maternal immunization against misfolded MSTN [20]. In chickens, the growth rate of chicks from hens immunized against MSTN was significantly lower than that of the control [19].

Currently, little is known about the reason behind the inconsistent results in the growth performance of offspring affected by maternal immunization against MSTN. Therefore, this study was aimed to investigate whether immunization of hens against MSTN renders anti-MSTN antibodies being transferred into fertilized eggs and the effects of *in-ovo* presence of anti-MSTN antibodies on post-hatch growth and skeletal muscle mass in chicken.

## Materials and methods

### Antigen production

#### Construction and transformation of chicken myostatin expression vector

Chicken myostatin (chMSTN) cDNA (S1 Fig) was commercially synthesized with codon optimization for *E*. *coli* expression and ligated into the pET-45b (+) plasmid at the BamHl, and Sall sites commercially (GenScript, PA, USA). The expression vector inserted with chMSTN (chMSTN-pET-45b) was transformed into the T7 Express *lysY E*. *coli* strain (New England Biolabs, MA, USA) following the manufacturer’s protocol. The transformants were spread on Luria-Bertani (LB) (1.0% tryptone, 0.5% yeast extract and 0.5% NaCl) agar plates containing 100 μg/mL ampicillin. Three colonies were selected, plasmids were purified and the inserted DNA sequence was analyzed to confirm correct insertion.

#### Expression of chMSTN protein

A selected colony was used to inoculate 5 mL LB medium with 100 μg/mL ampicillin for overnight growth at 37°C with vigorous shaking. The 5 mL cultures were used to inoculate 25 mL LB medium with 100 μg/mL ampicillin, and cells were grown to an OD_600_ of 0.4–0.6. To induce the expression of chMSTN protein, isopropyl-β-D-thiogalactoside (IPTG) was added to the culture to a final concentration of 0.2 mM, and the culture was grown at 37°C for 6 h under vigorous shaking at 250 rpm. The expression of chMSTN protein was examined using the SDS-PAGE analysis.

#### Large-scale production of chMSTN

Ten μL of glycerol stock of transformed *E*. *coli* was used to inoculate 10 mL of sterilized LB broth containing 100 μg of ampicillin per ml in a 50 mL tube. The culture was incubated overnight at 37°C with vigorous shaking at 250 rpm. The next day, 10 mL of the overnight culture was dispensed into 1 L autoclaved LB broth in a 2 L baffled flask and incubated at 37°C with vigorous shaking at 250 rpm, cells were grown to an OD_600_ of 0.4–0. 6. The cultures were then induced for protein expression for 4 h at 37°C with 0.2 mM of IPTG under vigorous shaking at 250 rpm. Upon induction, the cultures were centrifuged at 1,200 g. The media was removed, and cell pellets were weighed and suspended in 5–10 mL phosphate-buffered saline (PBS) (20 mM phosphate buffer containing 500 mM NaCl, pH 7.8) per gram of pellet. The suspension was incubated for 15 min after adding lysozyme (100 μg/mL, 2 μL of 50 mg/mL stock solution) and DNAse 1 (5 unit/mL, 2 μL of 2,500-unit/mL stock solution). The suspension was sonicated for 10 minutes with 15 s pulses to lyse the cells. Then, the tube was centrifuged for 30 min at 9,000 g. The pellet was washed twice using lysis buffer containing 50 mM potassium phosphate, 400 mM NaCl, 100 mM KCl, 10% glycerol, 0.5% Triton X-100, and 10 mM imidazole (pH 7.8). The pellet was further washed twice using distilled water to remove salts and detergents, and the final sediment containing the inclusion body was used for solubilization.

#### Isolation and solubilization of chMSTN inclusion body

One gram of inclusion body containing chMSTN was suspended in 8 mL commercially available inclusion body solubilizing solution (Thermo Scientific, Massachusetts, USA). A substantial amount of the recombinant chMSTN dissolved in this solution; thus, the supernatant was collected after centrifugation at 10,000 g for 20 min. The pooled solution (50 mL) containing solubilized chMSTN was dialyzed 5 times against a solution containing 0.25% (w/v) sodium bicarbonate, 0.2% (w/v) α-lactose, and 0.2% (w/v) mannitol, pH 8.5 [22]. The dialyzed solution was centrifuged at 10,000 g for 20 min to remove precipitating proteins. Protein concentration in the supernatant was determined by the Lowry method [23] using bovine serum albumin as a standard and stored at -20 °C for later use.

### Animal studies

#### Animal husbandry

All animal care procedures were approved by the Institutional Animal Care and Use Committee of the University of Hawaii (protocol#18–2961). Chickens were housed in the

Magoon Research Station Small Animal Facility, the University of Hawaii at Manoa. The temperature and humidity in the facility were controlled to maintain an appropriate climate for the chicken. One-week-old 12 Hy-Line Brown females and 4 White Leghorn males were obtained from a local hatchery and reared for 20 weeks with free access to water and feed. The one-week-old chicks were fed corn and soybean-based starter ration with 20.0% crude protein (CP) up to 6 weeks of age. From 6–12 weeks of age, they were offered corn and soybean-based grower ration with 19.0% CP. Between 12–18 weeks of age, they were fed corn and soybean-based developer ration with 18.0% CP. After 18 weeks of age, they were fed corn and soybean-based layer ration with 17.0% CP. The calcium contents of starter, grower, developer and layer were 1.01, 1.00, 2.06, and 4.10%, respectively. The male birds were offered the same diet as layers with a calcium level of 1%. At 18 weeks of age, hens were randomly allocated into 4-floor pens with a breeder’s feeding program plus freely available water, each pen with 3 hens and 1 rooster. The roosters were rotated among groups weekly to minimize the sire effect.

#### Immunization

**Fig 1:**
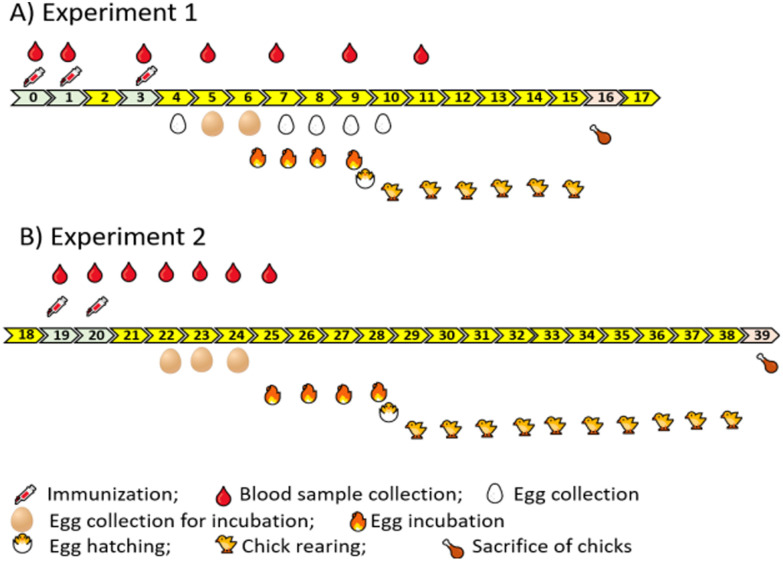
Animal experiment diagram from the first day of immunization. A) Experiment 1, B) Experiment 2. Numbers indicate weeks after the first immunization.

*Experiment 1*. At 24 weeks of age, the 4 floor pens were randomly divided into two groups with two pens in each group: Control and chMSTN. Hens in the chMSTN group were injected subcutaneously with 0.5 mg of chMSTN /100 μL of PBS emulsified with an equal volume of Titer Max adjuvant (Titer Max, GA, USA) on days 0, 7, and 21 (Fig 1A). Hens in the Control group were immunized with 100 μL of PBS emulsified with an equal volume of Titer Max adjuvant. Blood samples were collected from the brachial vein on the day of immunizations and every 2 weeks afterward for antibody titer measurement. The serum was separated from the clotted blood by centrifuging at 2,000 g for 7 min and stored at -20 °C for later analysis. Eggs were collected daily from the first day of immunization to measure the hen-day egg production and egg. Three eggs were randomly selected from each group between day 28 and day 70 from the first day of immunization for IgY extraction. Egg yolk IgY was extracted using a Chicken IgY Purification Kit (Thermo Scientific, IL, USA). The purity of extracted egg yolk IgY was confirmed by SDS-PAGE in reduced and non-reduced conditions.

*Experiment 2*. Sixteen weeks after the last immunization (43 weeks of age) in Experiment 1, immunization for the second experiment was performed (Fig 1B). In addition to chMSTN, a peptide fragment of the mature myostatin (Myo2) was also used as an antigen. The amino acid sequence of the Myo2 was VFLQKYPHTHLVHQA, representing amino acid sequences from 50 to 64 on the mature MSTN. Myo2 peptides were commercially synthesized and conjugated to keyhole limpet hemocyanin (KLH) (Global Peptide Services, CO, USA).

Birds from Experiment 1 were used in this experiment. Two floor pens of the Control group from the previous experiment remained as the Control group and one floor pen of the chMSTN group remained as the chMSTN group while one floor pen of the chMSTN group was assigned to the Myo2 group. The Control and chMSTN groups were immunized in the same way as Experiment 1 on days 0 and 7. The Myo2 group was immunized with 2 mg of Myo2 peptide in 100 μL of PBS emulsified with an equal volume of Titer Max adjuvant. Blood samples were collected from the brachial vein on the day of immunizations and every week post-immunization for antibody titer measurement. The serum was separated and stored at -20 °C for later analysis. Eggs were collected daily from the first day of immunization to measure the egg number and weight. One egg was randomly selected from each group on day 21 and day 28 from the first day of immunization for IgY extraction.

#### Post hatch chick’s growth

*Experiment 1*. After the confirmation of peak antibody titer in serum and egg yolk, about 60 eggs were collected from each group. Collected eggs were incubated under standard conditions. The hatchability of eggs from the Control and chMSTN groups were 77.5 and 84.4%, respectively. The chicks obtained after the hatching were reared for 7 weeks of age. The chicks were reared under a deep litter system. Each pen of 4 x 2 ft had about 6 chicks. They were fed type C medicated broiler starter ration with 22% protein (S1 Table, Purina Animal Nutrition LLC, MN, USA) for 4 weeks of age, followed by feeding type C medicated broiler finisher ration with 18% protein (S1 Table, Purina Animal Nutrition LLC, MN, USA). Bodyweight was measured weekly. No mortality was recorded during the experiment. The birds were sacrificed using carbon dioxide asphyxiation at 7 weeks of age, and breast and bone-in thigh muscles were separated and weighed.

*Experiment 2*. About thirty-three eggs were collected from each group between day 22 and day 33 from the first day of immunization. Collected eggs were incubated under standard conditions. The hatchability of eggs from the Control, chMSTN, and Myo2 groups were 77.5, 87.9, and 80.6%, respectively. The chicks obtained after the hatching were reared for 11 weeks of age. Chicks were fed in the same way as in Experiment 1. Bodyweight was measured weekly. No mortality was recorded during the experiment. At 11 weeks of age, birds were sacrificed using carbon dioxide asphyxiation, and skin, head, feet, and abdominal viscera were removed to measure the carcass weight. From carcasses, breast and bone-in thigh muscles were separated and weighed.

### SDS-PAGE

SDS-PAGE was performed with 12.5% polyacrylamide gels by Laemmli’s method [24]. One percent β-mercaptoethanol loading buffer (150 mM Tris-HCl, 30% glycerol, 3% SDS, 0.03% bromophenol blue, 10% 2-mercaptoethanol at pH 6.8) was mixed with the samples then incubated for 3–5 min at 100°C. Samples were then loaded in the polyacrylamide gels and subjected to electrophoresis, and gels were stained with Coomassie blue for visualization, followed by destaining.

### ELISA

Recombinant soluble chMSTN, or Myo2 conjugated to BSA (Myo2-BSA) was used as a coating antigen. 100 μL of soluble chMSTN (20 μg/mL) or Myo2-BSA (20 μg/mL) was added to each well of 96 well microtiter plates. The plate was incubated overnight at 4°C. The plates were blocked with 200 μL of 1% BSA after washing once with PBS. The plate was then incubated for two hours at room temperature, then washed twice with PBS. Diluted 100 μL chicken serums (1:2000) or egg yolk IgY solution (2.5 μg/mL) were added in triplicate, and the plates were incubated for two hours at room temperature. The horseradish peroxidase (HRP)-conjugated goat anti-chicken IgY (1:1000) (Abcam, MA, USA) was added, and then the plates were incubated for 2 h at room temperature. Following three washes with PBS containing 0.05% Tween-20, the 3,3´,5,5´-tetramethylbenzidine (TMB) substrate (Promega, WI, USA) was added to each well. After 20 min of incubation at room temperature, the absorbance at 405 nm was measured using a microplate reader (Bio-Rad, CA, USA).

### Western blot

Fractionated proteins in the gel were transferred to polyvinylidene difluoride (PVDF) membranes. The membrane was blocked with 3% BSA in Tween 20-Tris-buffered saline (TTBS) (0.1% Tween-20, Tris-buffered saline of 20 mM Tris-HCl, 150 mM NaCl at pH 7.5) with 0.1% sodium azide for 1 hour at room temperature. After discarding the blocking buffer, the membrane was incubated with 500 times diluted monoclonal anti MSTN antibody or 200 times diluted egg yolk IgY (2.1 mg/mL) in TTBS overnight at 4°C. The monoclonal anti-MSTN antibody was from a previous study (Kim et al, 2006). The membrane was then rinsed three times, with TTBS, followed by incubation with diluted (1:10,000) HRP-conjugated goat anti-mouse IgG antibody (Sigma-Aldrich, MO, USA) or diluted (1:1,000) HRP-conjugated goat anti-chicken IgY (Abcam, MA, USA) in TTBS for 1 h at room temperature. The membrane was then rinsed three times with TTBS, and HRP activity was detected using SuperSignal^™^ West Femto kit (Thermo Fisher Scientific, MA, USA) and images were captured by Invitrogen iBright^™^ CL1500 Imaging System (Thermo Fischer Scientific, MA, USA).

### pGL3 (CAGA)_12_-luciferase reporter assay

MSTN-inhibitory activities of serum and egg yolk IgY were examined by CAGA luciferase assay using HEK293 human embryonic kidney cells stably expressing pGL-(CAGA)_12_-luciferase gene construct [25]. 100 μL of cells (350,000 cells/mL) in DMEM containing 10% FBS, penicillin-streptomycin plus fungizone, and geneticin were seeded on a 96-well white culture plate and incubated for 24 h at 37°C with 5% CO_2_. Following the incubation, the medium was replaced with 100 μL serum-free DMEM. Then 1 nM MSTN (12.5 ng/mL, R&D Systems, MN, USA) plus various concentrations of IgY were added and incubated for 24 h at 37°C with 5% CO_2_. After incubation, the Bright-Glo luciferase substrate (Promega, WI, USA) was added and luminescence was measured using a microplate illuminometer (Synergy^™^ LX Multi-Mode Microplate Reader, Agilent, CA, USA). The following formula calculated the percentage inhibition of MSTN activity: percentage inhibition = (luminescence at 1 nM MSTN–luminescence at each ligand concentration) x 100 / (luminescence at 1 nM MSTN–luminescence at 0 nM MSTN).

### Statistical analysis

Data were analyzed by one-way or two-way ANOVA using Prism5 software (Graphpad, CA, USA). Means were compared by Tukey’s-HSD test.

## Results

### Antigen preparation

**Fig 2:**
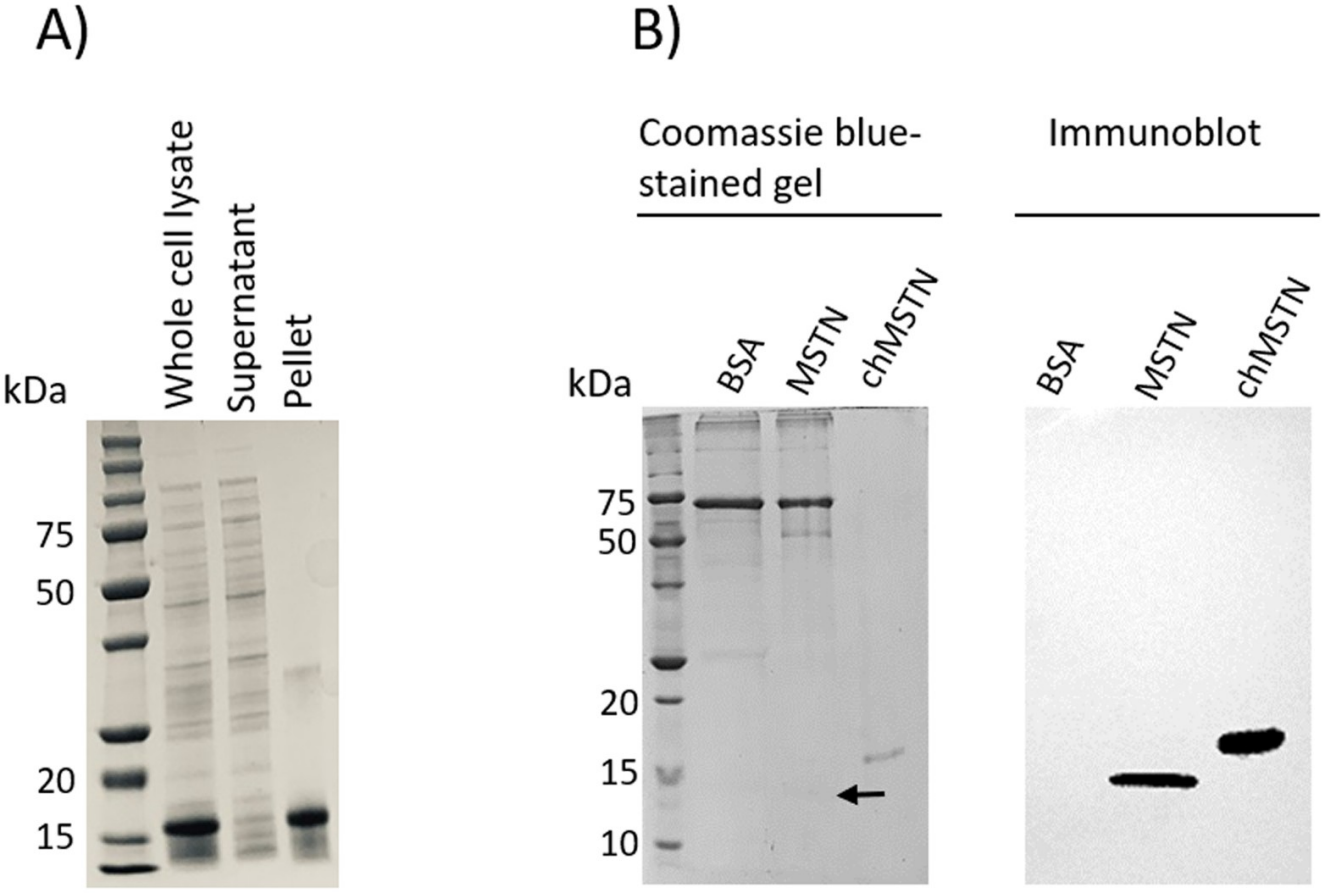
chMSTN antigen preparation. A) SDS-PAGE analysis of *E*. *coli* cell lysate after induction. The whole-cell lysate was centrifuged to separate supernatant and pellet, then the pellet was suspended in the same volume of PBS as the lysate volume. B) Western blot analysis of chMSTN. 300 ng of BSA (lane 2), 50 ng of commercial MSTN containing BSA as a carrier protein (lane 3), and 100 ng of solubilized chMSTN (lane 4) were subjected to SDS-PAGE and Western blot analysis. Monoclonal anti-MSTN antibody (0.34 mg/mL) was used as a primary antibody (1:500) and peroxidase-conjugated goat anti-mouse IgG (H+L) was used as a secondary antibody (1:20,000). The arrow indicates a faint MSTN band.

chMSTN (expected size of 16 kDa) was expressed as an inclusion body (Fig 2A). To confirm that the expressed protein is chicken MSTN, a Western blot was performed using a monoclonal antibody against MSTN. The monoclonal antibody showed a strong affinity to chMSTN as well as to a commercial MSTN (Fig 2B), confirming that the expressed protein is chicken MSTN.

### Antibody titers in immunized hen and egg yolk

#### Experiment 1

**Fig 3:**
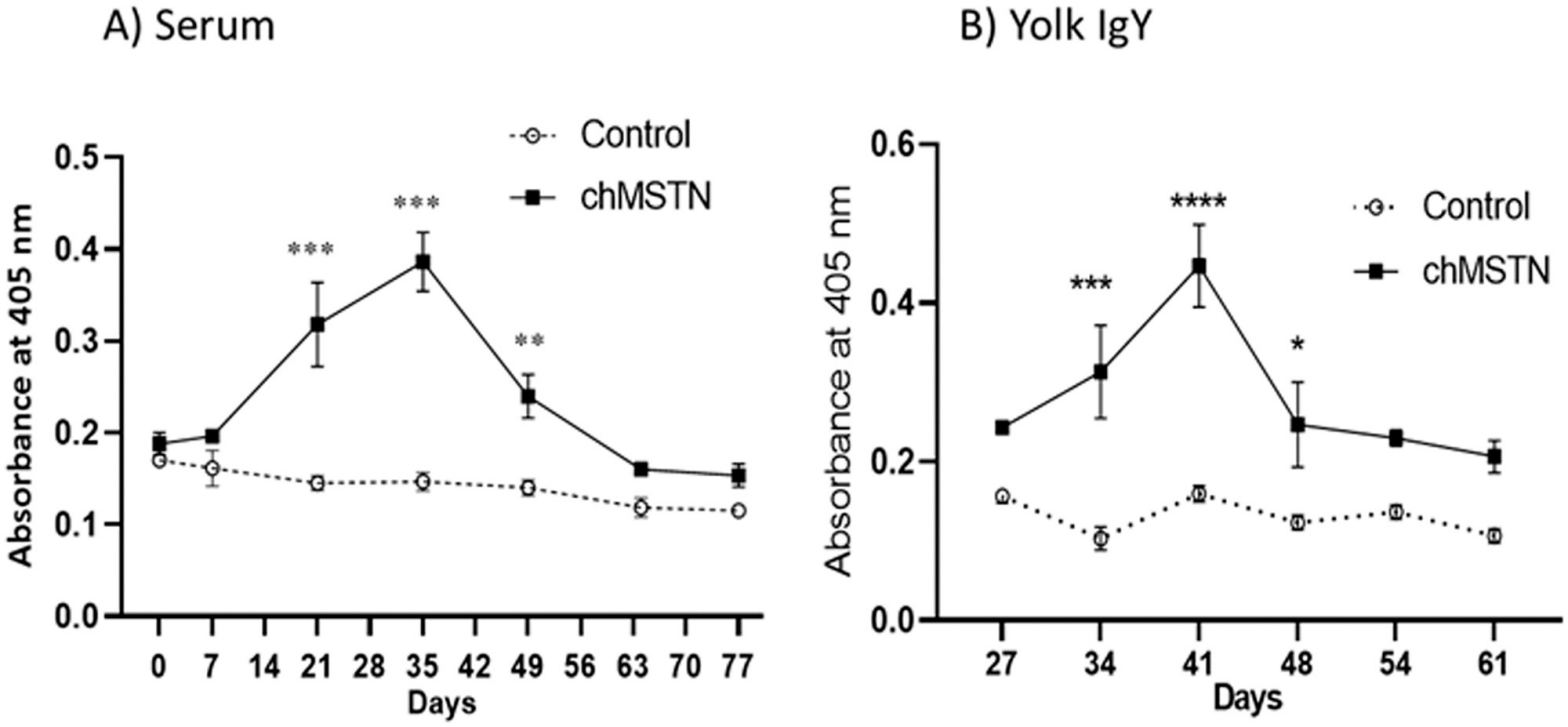
Titer dynamics of immunized hens and egg yolk against chMSTN. A) Diluted (1:2,000) serum from six hens of each group was used for titer determination. Arrowheads in the X-axis indicate the injection of antigen. B) 2.5 μg/mL egg yolk IgY from three hens of each group were used. The mean difference between the Control and chMSTN groups within days was analyzed by T-test. Data are mean ± SEM.

Hens’ serum titer values were gradually increased after immunization at 0, 7, and 21 days, reaching a peak at around day 35 from the first immunization (Fig 3A). No titer values were detected at 64 days after the first immunization. The titer values of yolk IgY against chMSTN increased gradually and reached the maximum on day 41 from the first immunization (Fig 3B), a week after the peak serum antibody titer.

#### Experiment 2

**Fig 4:**
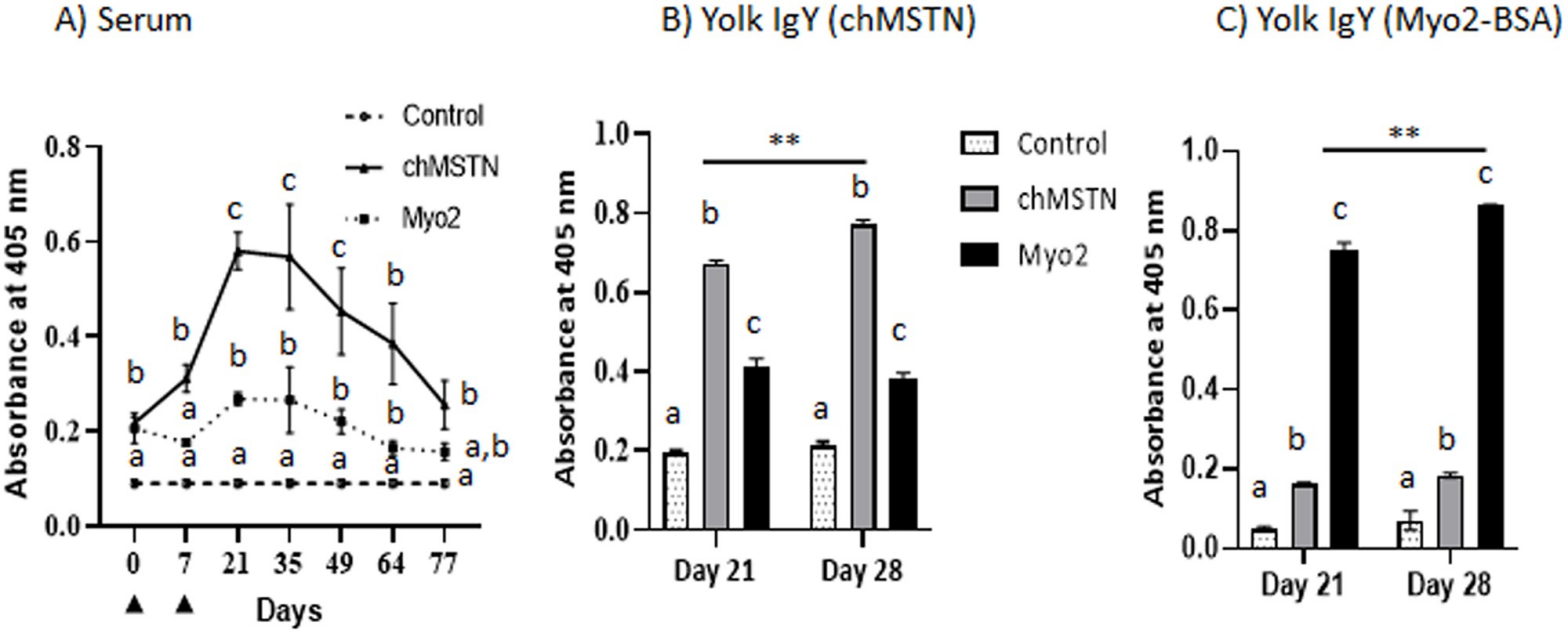
Antibody titer dynamics of immunized hens and egg yolk against chMSTN. A) Diluted (1:2,000) serum was used for titer determination. Serums were collected from the Control (n = 6), chMSTN (n = 3), and Myo2 (n = 3) hens, respectively. Arrowheads in the X-axis indicate the injection of antigen. Data are mean ± SEM. 2.5 μg/mL egg yolk IgY from three hens of each group were used for titer measurement with chMSTM (B) or Myo2 conjugated to BSA (C) as a coating antigen. The mean difference among the three groups within days and the mean difference between days 21 and 28 within treatment groups were analyzed by Tukey’s test. Different alphabets within the days are different at P<0.05. Data are mean ± SEM. **, P<0.01.

The peak serum titer was seen around day 21 from the day of the first immunization (Fig 4). The titer values of the Myo2 group were lower than those of the chMSTN group probably due to the use of chMSTN as a coating antigen (Fig 4A). Antibodies in egg yolk was detected at 21 and 28 days after the first immunization. The IgY from eggs of the chMSTN group on days 21 and 28 showed a significantly higher amount of chMSTN-specific antibodies than those of the Myo2 group (Fig 4B). When Myo2-BSA was used as coating antigen, IgY from Myo2-KLH immunized birds showed significantly higher antibody titer than those of the Control and chMSTN groups (Fig 4C).

### Western blot analysis of the MSTN-binding characteristics of IgY from chMSTN and Myo2 groups

**Fig 5:**
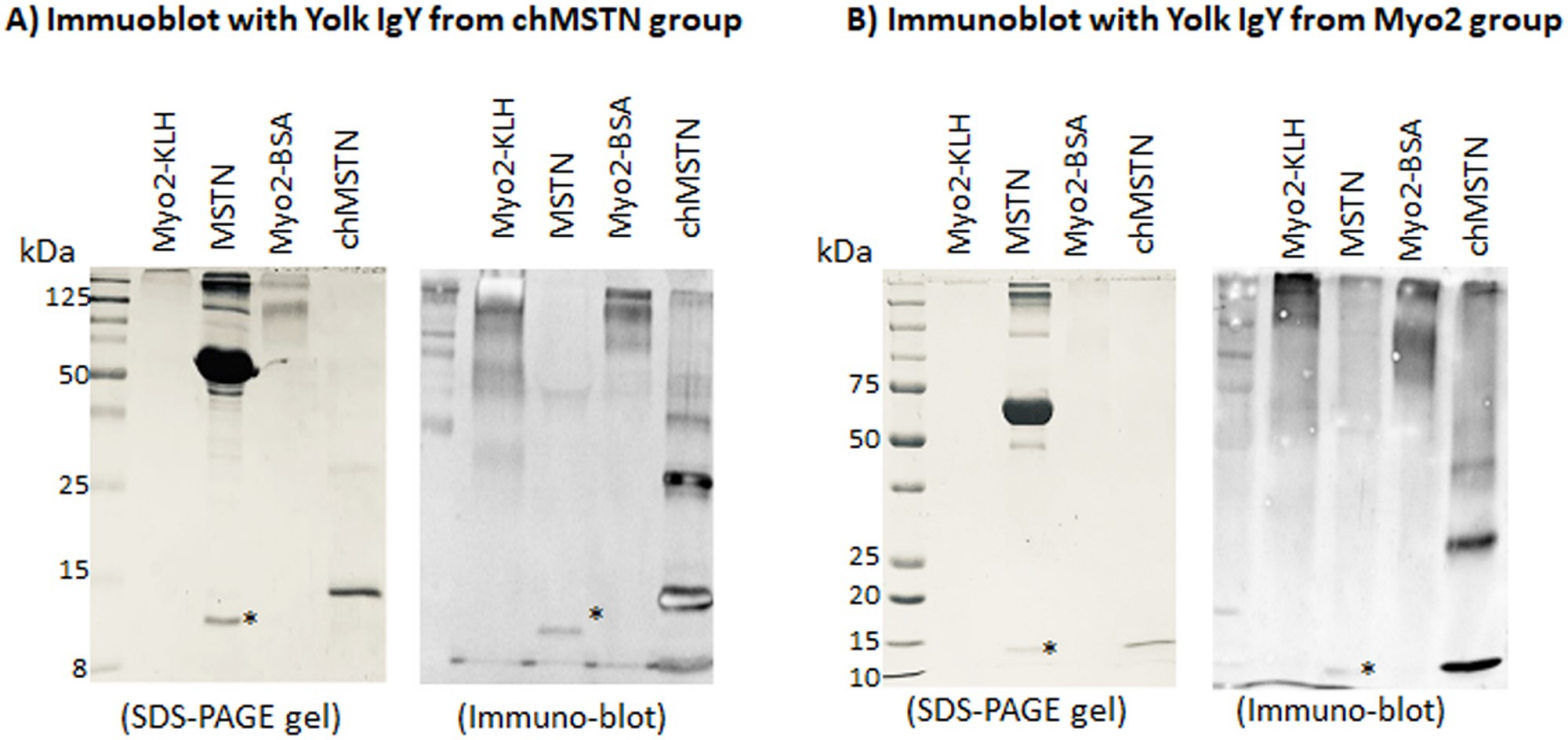
Western blot analysis of binding characteristics of egg yolk IgY from the chMSTN and Myo2 groups. 400 ng of Myo2-KLH, Myo2-BSA, chMSTN, and 200 ng of MSTN with BSA as a carrier protein were subjected to SDS-PAGE, and Western blot was performed using 1:200 dilution of egg yolk IgY (2.1 mg/mL) from the chMSTN group (A) or the Myo2 group (B) as a primary antibody, 1:10,000 diluted HRP conjugated anti-chicken IgY as a secondary antibody, and luminescence substrate for signal detection. The asterisks indicate MSTN band.

The binding characteristics of IgY from the chMSTN and Myo2 groups to MSTN was examined using Western blot analysis. The egg yolk IgY from the chMSTN and Myo2 groups showed an affinity to MSTN, chMSTN, and Myo2-KLH and Myo2-BSA with the strongest affinity to their immunogen based on visible band intensity (Fig 5). The IgY from the Myo2 group (immunized against Myo2-KLH) showed the affinity to Myo2-BSA, indicating that immunization against Myo2-KLH produced IgY against Myo2 peptide as well as against KLH. The affinity of egg yolk IgY from both the chMSTN and Myo2 groups to commercial MSTN appeared to be less than its affinity to chMSTN, Myo2-KLH, or Myo2-BSA based on the band intensity in the immunoblot (Fig 5), suggesting that hen immunized against chMSTN or Myo2 produced weak antibody against MSTN.

### Analysis of MSTN-inhibitory capacities of IgYs from the chMSTN and Myo2 groups

**Fig 6:**
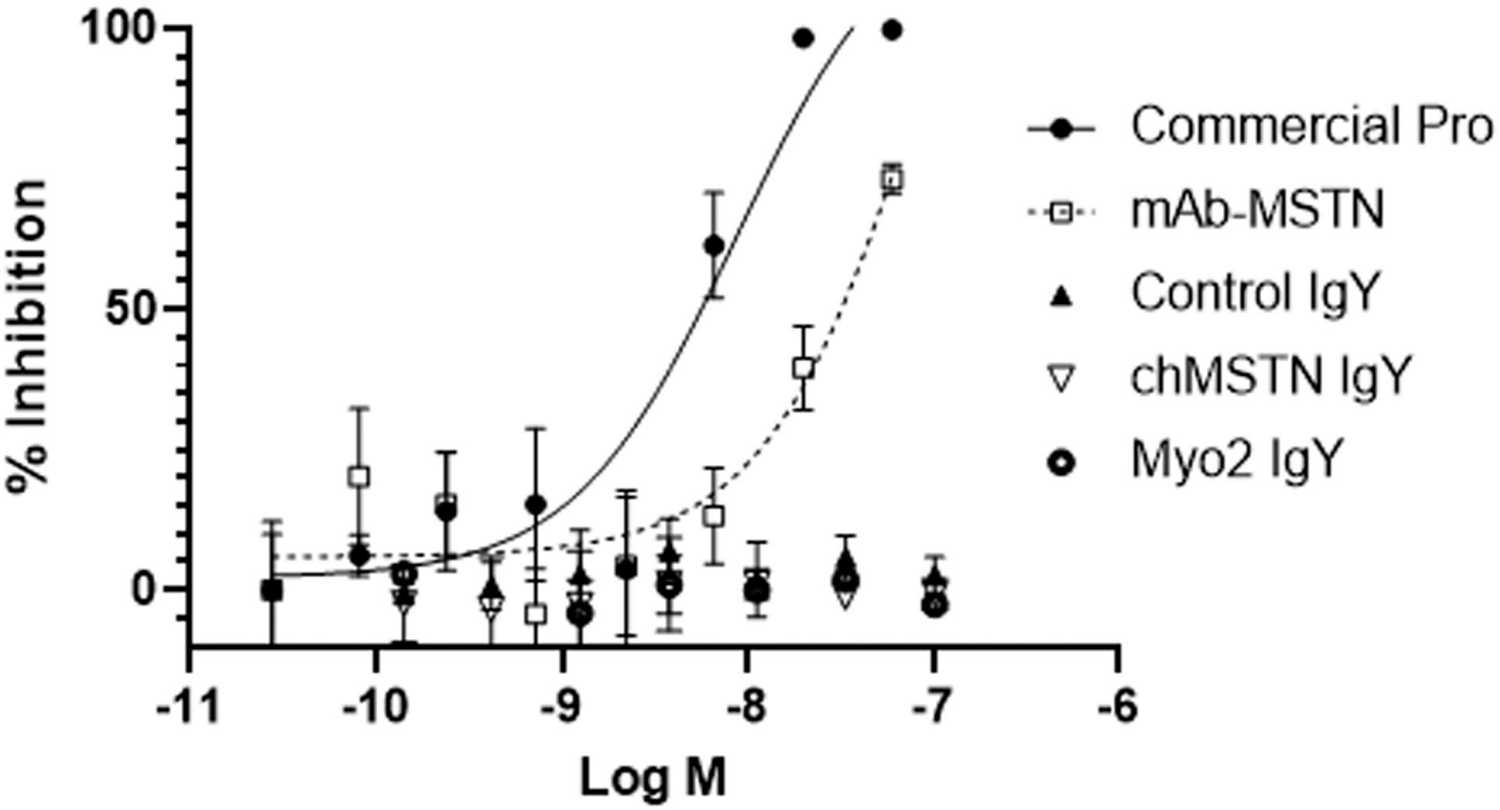
Inhibition of MSTN activity by egg yolk IgY. HEK293 cells stably expressing (CAGA)_12_- luciferase gene construct were seeded on a 96-well culture plate at 35,000 cells/well and grown for 24 h in DMEM with 10% fetal calf serum, antibiotics, and antimycotic. The medium was removed, and various concentrations of commercial MSTN propeptide (Commercial Pro), monoclonal anti-MSTN antibody (mAb-MSTN), egg yolk IgYs from the Control, chMSTN, and Myo2 groups in DMEM containing 1 nM MSTN were added to each well, followed by incubation for 24 hr. The medium was removed, luminescence substrate was added, followed by luminescence measurement. The % inhibition of MSTN activity was calculated by the following formula: % inhibition = (luminescence at 1 nM MSTN—luminescence at each ligand concentration) *100/(luminescence at 1 nM MSTN—luminescence at 0 nM MSTN). The error bars represent the mean ± SEM (n = 3).

The MSTN-inhibitory capacities of IgYs from the chMSTN and Myo2 groups were examined using the pGL3(CAGA)_12_-luciferase reporter system (Fig 6). Both MSTN propeptide and monoclonal anti-MSTN antibody demonstrated MSTN-inhibitory capacity with commercial MSTN propeptide having higher MSTN-inhibitory capacity (IC_50_ for 1 nM MSTN, 8.6 nM) than monoclonal anti-MSTN antibody (IC_50_ for 1 nM MSTN, 96.1 nM). However, the IgYs from the chMSTN or Myo2 groups did not show MSTN-inhibitory capacity, indicating that the IgYs from the chMSTN or Myo2 groups could not to inhibit the biological activity of MSTN in the concentration range tested (27 pM to 60 nM).

### Effect of maternal immunization against chMSTN on the bodyweights of offspring

#### Experiment 1

**Fig 7:**
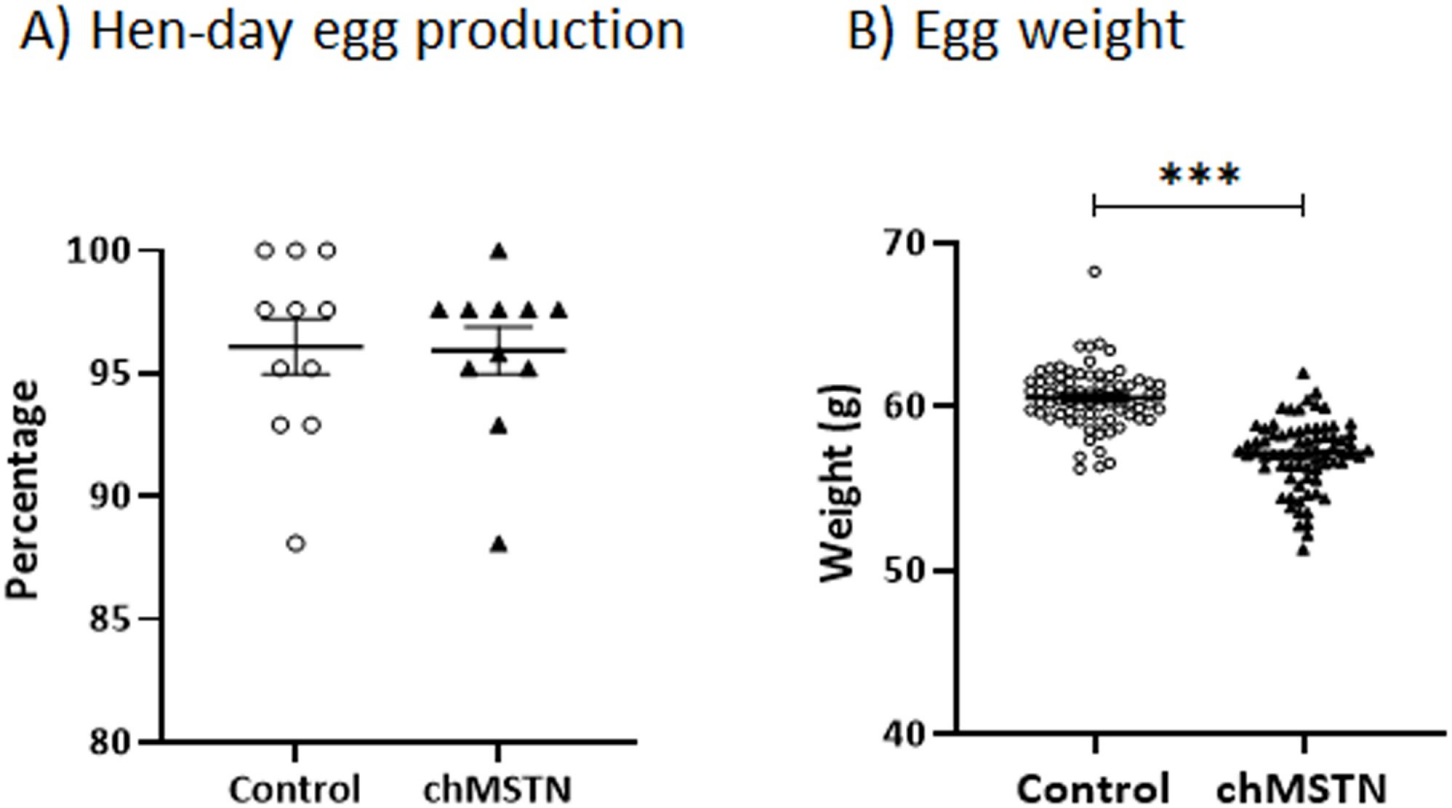
Hen-day egg production (A) and egg weights (B) in Experiment 1. Hen-day egg production was calculated by dividing the number of eggs produced within a week period by the average number of hens for the same time interval during the 11 weeks. Data were analyzed by one-way ANOVA. ***, P<0.001.

*Egg production and weights*. There was no significant difference in hen day egg production between the Control and chMSTN groups (Fig 7A), but the egg weight of the chMSTN group (57.1±0.30 gm) was significantly lower than that of the control group (60.5 ± 0.21 gm) (Fig 7B).

*Body weights and carcass characteristics of chicks from the Control and chMSTN groups*. No difference was found in the bodyweight of chicks between the Control and chMSTN groups regardless of sex during the trial period (Table 1). No significant difference was also found in carcass parameters including breast muscle, bone-in thigh muscle, liver, and heart weights between the Control and chMSTN groups (Table 2). Even though the egg weight of the chMSTN group was lower than that of the Control group, the bodyweight of the chMSTN group was not different from that of the Control group, indicating that the egg weight did not affect the post-hatch growth of chicks.

**Table 1:** Body weight of chicks from the Control and chMSTN groups in Experiment 1.

Days	Treatment		Sex		Significance		
	Control (48)	chMSTN (51)	Male (42)	Female (57)	Trt	Sex	Int
7	74 ±1.2	72 ±1.0	75 ±0.4	72 ±1.8	NS	*	NS
14	124 ±2.2	123 ±2.0	128 ±1.4	118 ±2.1	NS	**	NS
21	205 ±3.4	205 ±3.5	217 ±2.5	194 ±3.5	NS	***	NS
28	285 ±4.2	282 ±5.4	301 ±4.2	266 ±4.4	NS	***	NS
35	391 ±6.2	376 ±6.1	411 ±6.0	356 ±6.3	NS	***	NS
42	514 ±7.7	509 ±7.5	556 ±7.4	467 ±7.7	NS	***	NS
49	764 ±11.5	767 ±11.3	853 ±11.1	677 ±11.6	NS	***	NS

Control group, 23 males and 25 females; chMSTN group, 29 males and 22 females. All the weight is in grams. Trt, treatment; Int, interaction; NS, non-significant. Data were analyzed by two-way ANOVA. Data are least-square means ± SEM.

*, P<0.05;

**, P<0.01;

***, P<0.001.

**Table 2:** Carcass characteristics of chicks from the Control and chMSTN groups in Experiment 1.

Item	Treatment		Sex		Significance		
	Control (48)	chMSTN (51)	Male (42)	Female (57)	Trt	Sex	Int
FBW	764 ± 11.5	767 ± 11.3	853 ± 11.1	677 ± 11.6	NS	***	NS
BM	105 ± 2.2	103 ± 2.6	115 ± 1.9	94 ± 2.0	NS	***	NS
TM	134 ± 2.4	135 ± 2.3	154 ± 2.3	115 ± 2.4	NS	***	NS
HW	4.0 ± 0.07	3.9 ± 0.07	4.5 ± 0.07	3.4 ± 0.07	NS	***	NS
LW	19.3 ± 0.47	19.3 ± 0.46	21.6 ± 0.46	17.0 ± 0.48	NS	***	NS

Control group, 23 males and 25 females; chMSTN group, 29 males and 22 females. FBW, final body weight; BM, breast muscle weight; TM, bone-in thigh muscle weight; HW, heart weight; LW, liver weight. Trt, treatment; Int, interaction; NS, non-significant. Data were analyzed by two-way ANOVA. Data are least-square means ± SEM.

***, P<0.001.

#### Experiment 2

**Fig 8:**
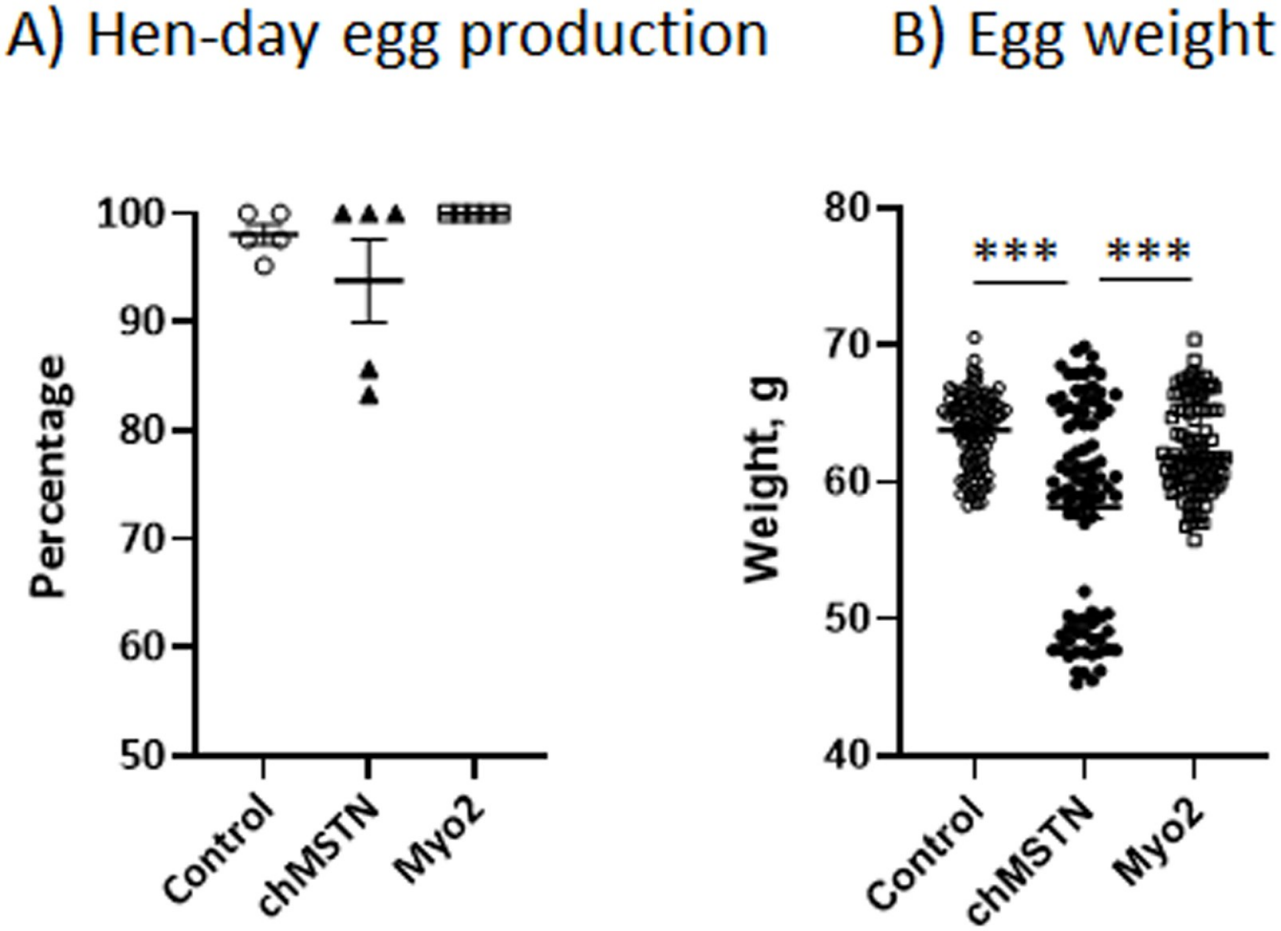
Hen-day egg production (A) and egg weight (B) in Experiment 2. Hen day egg production was calculated by dividing the number of eggs produced within a week by the average number of hens on hand for the same time interval during the 5 weeks. Data were analyzed by one-way ANOVA. Means were compared by Tukey’s-HSD test. ***, P<0.001.

*Egg production and weights*. There was no significant difference in hen day egg production among the Control, chMSTN, Myo2 groups (Fig 8A). The egg weight of chMSTN group (58.2 ± 0.80 gm) was significantly lower than the Control (63.8 ± 0.28 gm) or Myo2 groups (62.4 ± 0.34 gm) (Fig 8B).

*Body weights and carcass characteristics of chicks from the Control*, *chMSTN*, *and Myo2 groups*. No significant difference was found in the body weight between the Control and chMSTN groups, while the body weight of the Myo2 group was significantly lower than that of the Con and chMSTN groups, regardless of sex during the feeding trial (Table 3). The final body weight of the Myo2 group was 8.4% and 5.7% less than that of the Control and chMSTM groups, respectively.

**Table 3:** Bodyweights of chicks from the Control, chMSTN, and Myo2 groups in Experiment 2.

Days	Treatment			Sex		Significance		
	Control (23)	chMSTN (32)	Myo2	Male	Female	Trt	Sex	Int
			(33)	(42)	(46)			
7	92^a^	90^a^	81^b^	90	85	***	**	NS
	(1.5)	(1.3)	(1.3)	(1.2)	1.1			
14	164^a^	163^a^	150^b^	168a	150	**	***	NS
	(3.2)	(3.7)	(2.7)	(2.4)	(2.3)			
21	258^a^	256^a^	240^b^	270	232	**	***	NS
	(5.1)	(4.3)	(4.3)	(3.8)	(3.7)			
28	370^a^	367^a^	343^b^	390	331	**	***	NS
	(7.8)	(6.4)	(6.3)	(5.7)	(5.4)			
35	507 ^a^	501 ^a^	461^b^	538	441	***	***	NS
	(9.6)	(8.2)	(8.0)	(7.2)	(6.9)			
42	631^a^	631^a^	578^b^	677	550	***	***	NS
	(11.6)	(9.8)	(9.7)	(8.7)	(8.3)			
49	766^a^	762^a^	705^b^	829	660	***	***	NS
	(14.1)	(11.9)	(11.7)	(10.5)	(10.1)			
56	909^a^	892^a^	839^b^	985	775	**	***	NS
	(16.9)	(14.3)	(14.1)	(12.7)	(12.1)			
63	1074^a^	1035^a^	962 ^b^	1153	895	***	***	NS
	(17.6)	(15.0)	(14.7)	(13.2)	(12.6)			
70	1196^a^	1161^a^	1066^b^	1290	992	***	***	NS
	(18.7)	(15.9)	(15.6)	(14.0)	(13.4)			
77	1351^a^	1312^a^	1237^b^	1481	1119	***	***	NS
	(22.0)	(18.7)	(18.3)	(16.5)	(15.7)			

Control group,11 males and 12 females; chMSTN group, 15 males and 17 females; Myo2 group, 16 males and 17 females. All the weight is in grams. Data were analyzed by two-way ANOVA. Means were compared by Tukey’s-HSD test. Data are least-square means (SEM).

**, P<0.01;

***P<0.001.

Means not sharing the same superscript differ at P<0.05. Trt, treatment; Int, interaction; NS, non-significant.

Table 4 summarizes the carcass characteristics of chicks from the Control, chMSTN, and Myo2 groups. The carcass weight of the Myo2 group was significantly lower than that of the Control and chMSTN groups with no significant difference between the Control and chMSTN groups. The dressing % of chMSTN and Myo2 groups were smaller than that of the Control group with no difference between the chMSTN and Myo2 groups. The breast muscle weight of the Myo2 group was significantly smaller than those of the chMSTN (9.2%) and Control groups (16.5%) and the weight of chMSTN group was significantly smaller than that of the Control group. The percentage of breast muscle to body weight of the Myo2 group was significantly smaller than those of the chMSTN and Control groups with no difference between the chMSTN and Control groups. The bone-in thigh muscle weight of the Myo2 group was also significantly smaller (10.7%) than that of the Control group. In contrast to Experiment 1, the breast muscle weight, and its percentage to the body weight of the chMSTN group was significantly lower than those of the Con group. The growth period differed between Experiment 1 (7 weeks) and 2 (11 weeks). Close examination of the growth data of Experiment 2 shows that the body weights of the Control and chMSTN groups were similar up to 7 weeks of age, and afterward, the body weight of chMSTN group tended to be smaller than that of the Control group, suggesting that maternal immunization of hens against chMSTN potentially negatively affected the later stage of growth of offspring.

**Table 4:** Carcass characteristics of chicks from control, chMSTN, and Myo2groups in Experiment 2.

Items	Treatment			Sex		Significance		
	Control	chMSTN	Myo2	Male	Female	Trt	Sex	Int
	(23)	(32)	(33)	(42)	(46)			
FBW	1351^a^	1312^a^	1237 ^b^	1481	1119	***	***	NS
	(22.0)	(18.7)	(18.3)	(16.5)	(15.7)			
CW	714^a^	679 ^a^	639 ^b^	776	579	***	***	NS
	(13.4)	(11.3)	(11.1)	(10.0)	(9.6)			
DP	52.7 ^a^	51.6 ^b^	51.7 ^b^	52.3	51.7	*	NS	NS
	(0.68)	(0.38)	(0.15)	(0.97)	(0.43)			
BM	188^a^	173^b^	157^c^	190	155	***	***	NS
	(5.8)	(5.4)	(3.2)	(3.0)	(2.9)			
%BM	14^a^	13.2^b^	12.8^b^	12.8	13.9	***	***	NS
	(0.18)	(0.15)	(0.15)	(0.13)	(0.13)			
TM	262^a^	248^ab^	234^b^	291	204	***	***	NS
	(11.3)	(9.4)	(7.5)	(11.4)	(4,5)			
%TM	19.3^a^	18.8^a^	18.9^a^	19.7	18.3	NS	***	NS
	(0.17)	(0.15)	(0.14)	(0.13)	(0.12)			

Control group,11 males and 12 females; chMSTN group, 15 males and 17 females; Myo2 group, 16 males and 17 females. Data are least-square means (SEM)***P<0.001; **, P<0.01; *, P<0.05. Means were compared by Tukey’s-HSD test. Means not sharing the same superscript differ at P<0.05. All the weight is in grams (g) and age is in days. FBW, final body weight; CW, carcass weight; BM, breast muscle weight; %BM, percent breast muscle; TM, bone-in thigh muscle weight; %TM, percent thigh muscle. Trt, treatment; Int, interaction; NS, non-significant.

## Discussion

In a previous study, we observed that *in-ovo* administration of monoclonal anti-MSTN antibodies in the yolk significantly improved post-hatch body and muscle weights in broilers [9], indicating the potential of improving chicken growth performance by immunoneutralization of MSTN. However, antibody administration to fertilized eggs would not be easy for industry application. It is well known that antibodies are taken up into the egg yolk from hens’ blood and transferred into the embryonic circulation [26–28], thus active immunization of hens against MSTN would be an alternative approach to deliver anti-MSTN antibodies to fertilized egg yolks in chicken.

The current results from ELISA and Western blot showed a generation of IgY against MSTN and their presence in egg yolk when hens were immunized against misfolded MSTN (chMSTN) or synthetic MSTN fragment (Myo2). The hypothesis was that the anti-MSTN IgY present in egg yolk would potentially suppress MSTN activity during the embryonic and early post-hatch growth, leading to enhanced muscle growth of post-hatch chicks. However, contrary to the expectation, the current study showed that maternal immunization against chMSTN did not affect the growth performance and muscle mass of chicks in Experiment 1 and decreased the muscle mass in Experiment 2. When Myo2 was used as an antigen in Experiment 2, the growth performance and muscle mass of chicks were suppressed. The suppression of muscle in the Myo2 group was significantly greater than that of the chMSTN group. The egg weight of the Myo2 group was not different from that of the Control group but significantly smaller than that of the chMSTN group, thus it is unlikely that egg weight affected the body weight and muscle mass. Previous studies generally examined the binding capacities of anti-MSTN IgY to MSTN but did not examine the biological capacity of anti-MSTN IgY to suppress MSTN activity, thus we examined the MSTN-suppressive capacity of yolk IgYs using a pGL3 (CAGA)_12_ Luciferase reporter assay. The yolk IgYs from hens immunized against chMSTN or Myo2 showed little MSTN-inhibitory capacity, while the monoclonal anti-MSTN antibody that was used in *in-ovo* administration study exhibited a strong MSTN-inhibitory capacity comparable to that of MSTN propeptide (Fig 1), indicating that not all anti-MSTN antibodies can suppress the bioactivity of MSTN. This result partially explains why active immunization of hens against MSTN did not enhance body weight growth and muscle mass of post-hatch chicks.

Currently, the reason for the decrease in body weight and muscle mass of offspring from immunized hens against MSTN, particularly with Myo2, cannot be clearly explained. Similar to our results, a previous study also observed that maternal immunization against MSTN reduced the body weight of chicks from the immunized hens [19]. The study also showed that the extent of the decrease in body weight was positively related to titer levels. In this regard, it is tempting to speculate that the anti-MSTN antibodies from hens immunized against MSTN potentiated the MSTN activity, leading to suppression of muscle growth. It is known that MSTN regulates the generation and hypertrophy of type IIB muscle fiber more than other muscle fiber types, and studies have shown that MSTN suppression increased the number of type IIB muscle fibers [29] while overexpression of MSTN reduced type IIB muscle fiber type [30]. The current data showed that the breast muscle that is composed of exclusively type IIB muscle fiber in chicken [31, 32] was significantly reduced in chicks from hens immunized with Myo2, supporting the potential of the IgY to potentiate the MSTN activity. This study used layer chickens with broiler diets for post-hatch growth of chicks to provide enough protein needed for enhanced muscle growth induced by MSTN suppression. Apparently, enhanced muscle growth did not happen in chicks from hens immunized against MSTN. Since layers are a slow-growing breed, it is questioned whether the diet was a possible factor affecting the growth performance. However, as discussed previously, the growth of chicks from broiler hens immunized against MSTN was suppressed [19]. Thus, it is unlikely that the diet affected the growth performance of chicks. It should be noted that maternal immunization against MSTN improved body weight or muscle mass in mice [20, 21]. Even though limited in number, these studies suggest that the effect of maternal immunization on the body weight and muscle mass of offspring can be different among species.

In summary, our results show that the anti-MSTN antibodies generated by immunization of hens against chicken MSTN or MSTN fragment were transferred to egg yolk but the antibodies had no MSTN-inhibitory capacity. The maternal immunization against MSTN did not increase but decreased the body weight and muscle mass of chicks from the immunized hens, suggesting that the anti-MSTN antibodies potentially enhanced the MSTN activity instead of suppressing MSTN activity. The results, thus, bring an interesting insight that maternal immunization against certain molecules can affect the body weight and muscle mass of offspring.

## Supporting information

S1 Table Feed composition. (PDF)

S1 Fig DNA sequence of chMSTN optimized for E. coli expression. (PDF)

S2 Fig Western blot results. (PDF)

S3 Fig SDS-PAGE (7%) analysis of monoclonal anti-MSTN antibody (mAb), egg yolk IgY and serum in non-reduced condition. (PDF)
