# Supplementary material for: Maternal immunization against myostatin suppresses post-hatch chicken growth
Source: PLoS One. 2022 Oct 6;17(10):e0275753. doi: 10.1371/journal.pone.0275753 (PMC9536644; doi:10.1371/journal.pone.0275753)
Supplement: S1 Table — (PDF) [file pone.0275753.s001.pdf]

## S2. Feed composition

**Breeder hen feed**

| **Ingredients** | **Starter (0-6 wks)** | **Grower (6-12 wks)** | **Developer (12-18 wks)** | **Layer (18 wks onwards)** |
|---|---|---|---|---|
| Corn | 57.10 | 59.37 | 59.79 | 51.51 |
| SBM | 34.50 | 32.60 | 29.50 | 28.50 |
| Soybean oil | 3.64 | 3.64 | 1.00 | 5.00 |
| Limestone | 1.50 | 1.50 | 4.20 | 9.50 |
| Monocalcium phosphate | 1.80 | 1.75 | 1.90 | 1.90 |
| Lysine | 0.14 | 0.00 | 0.00 | 0.00 |
| Methionine | 0.25 | 0.20 | 0.16 | 0.14 |
| Threonine | 0.10 | 0.00 | 0.00 | 0.00 |
| Salt (NaCl) | 0.35 | 0.32 | 0.33 | 0.33 |
| Sodium bicarbonate | 0.12 | 0.12 | 0.12 | 0.12 |
| Vitamin + mineral mix | 0.50 | 0.50 | 0.50 | 0.50 |
| Total | 100.00 | 100.00 | 100.00 | 100.00 |

**Broiler starter/grower AMP medicated. Type C medicated feed.**

| **Nutrient** | **Min/Max** | **Amount** |
|---|---|---|
| Crude Protein | Min | 22.00 % |
| Lysine | Min | 1.00 % |
| Methionine | Min | 0.45 % |
| Crude Fat | Min | 3.50 % |
| Crude Fiber | Max | 4.00 % |
| Calcium (Ca) | Min | 0.90 % |
| Calcium (Ca) | Max | 1.40 % |
| Phosphorus (P) | Min | 0.60 % |
| Salt (NaCl) | Min | 0.30 % |
| Salt (NaCl) | Max | 0.80 % |
| Amprolium | | 0.0125% |

Manufactured by Purina Animal Nutrition LLC.

**Hawaiian grain® south pacific broiler finisher medicated. Type C medicated feed**

| Nutrient | Min/Max | Amount |
|---|---|---|
| Crude Protein | Min | 18.00% |
| Lysine | Min | 0.86% |
| Methionine | Min | 0.40% |
| Crude Fat | Min | 3.50% |
| Crude Fiber | Max | 5.00% |
| Calcium (Ca) | Min | 0.90% |
| Calcium (Ca) | Max | 1.40% |
| Phosphorus (P) | Min | 0.50% |
| Salt (NaCl) | Min | 0.20% |
| Salt (NaCl) | Max | 0.70% |
| Amprolium | | 0.0125% |

Manufactured by Purina Animal Nutrition
