# Supplementary material for: Maternal immunization against myostatin suppresses post-hatch chicken growth
Source: PLoS One. 2022 Oct 6;17(10):e0275753. doi: 10.1371/journal.pone.0275753 (PMC9536644; doi:10.1371/journal.pone.0275753)
Supplement: S1 Fig — (PDF) [file pone.0275753.s002.pdf]

S1. DNA sequence of chMSTN optimized for E. coli expression

CGT AGC CGT CGT GAC TTC GGT CTG GAC TGC GAT GAG CAC AGC ACC GAA AGC CGT TGC TGC CGT
R S R R D F G L D C D E H S T E S R C C R
TAC CCG CTG ACC GTG GAC TTC GAG GCG TTT GGT TGG GAT TGG ATC ATT GCG CCG AAG CGT TAC
Y P L T V D F E A F G W D W I I A P K R Y
AAA GCG AAC TAT TGC AGC GGC GAG TGC GAG TTC GTG TTT CTG CAG AAG TAT CCG CAC ACC CAC
K A N Y C S G E C E F V F L Q K Y P H T H
CTG GTT CAC CAA GCG AAC CCG CGT GGT AGC GCG GGT CCG TGC TGC ACC CCG ACC AAA ATG AGC
L V H Q A N P R G S A G P C C T P T .K Met S
CCG ATC AAC ATG CTG TAC TTT AAC GGC AAG GAA CAG ATC ATT TAT GGC AAA ATT CCG GCG ATG
P I N Met L Y F N G K E Q I I Y G K I P A Met
GTG GTT GAT CGT TGC GGC TGC AGC TAA
V V D R C G C S Stop

The DNA sequence of the expression plasmid was verified by sequence analysis (Advanced Studies in Genomics, Proteomics, and Bioinformatics (ASGPB), University of Hawaii at Manoa).
