# Supplementary material for: Maternal immunization against myostatin suppresses post-hatch chicken growth
Source: PLoS One. 2022 Oct 6;17(10):e0275753. doi: 10.1371/journal.pone.0275753 (PMC9536644; doi:10.1371/journal.pone.0275753)
Supplement: S2 Fig — (PDF) [file pone.0275753.s003.pdf]

S3. Western blot results

MSTN and chMSTN blot with monoclonal anti-MSTN antibody (Fig 2 in manuscript)

1 2 3 4

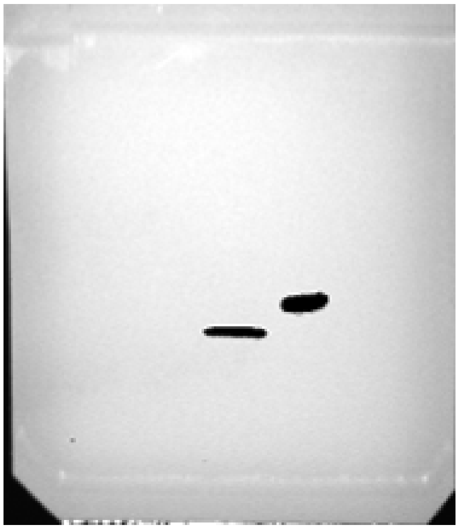

Lanes 1, 2, 3, and 4 indicate standard, BSA, MSTN and chMSTN

Immuno-blot with yolk IgY from the chMSTN group (Fig 5A in manuscript)

1 2 3 4 5

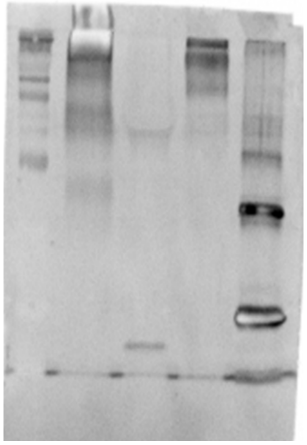

Lanes 1, 2, 3, 4, and 5 indicate standard, Myo2-KLH, MSTN, Myo2-BSA, and chMSTN

Immunoblot with yolk IgY from the Myo2 group (Fig 5B in manuscript)

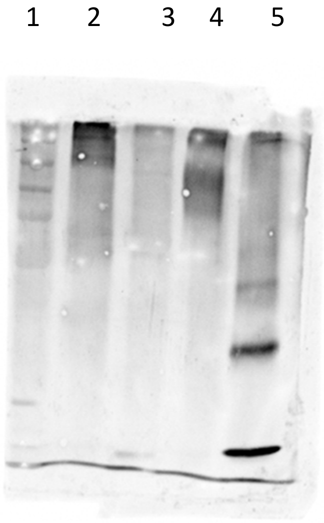

Lanes 1, 2, 3, 4, and 5 indicate standard, Myo2-KLH, MSTN, Myo2-BSA, and chMSTN
