# Supplementary material for: Maternal immunization against myostatin suppresses post-hatch chicken growth
Source: PLoS One. 2022 Oct 6;17(10):e0275753. doi: 10.1371/journal.pone.0275753 (PMC9536644; doi:10.1371/journal.pone.0275753)
Supplement: S3 Fig — (PDF) [file pone.0275753.s004.pdf]

**S4. SDS-PAGE (7%) analysis of monoclonal anti-MSTN antibody (mAb), egg yolk IgY, and serum in non-reduced condition**

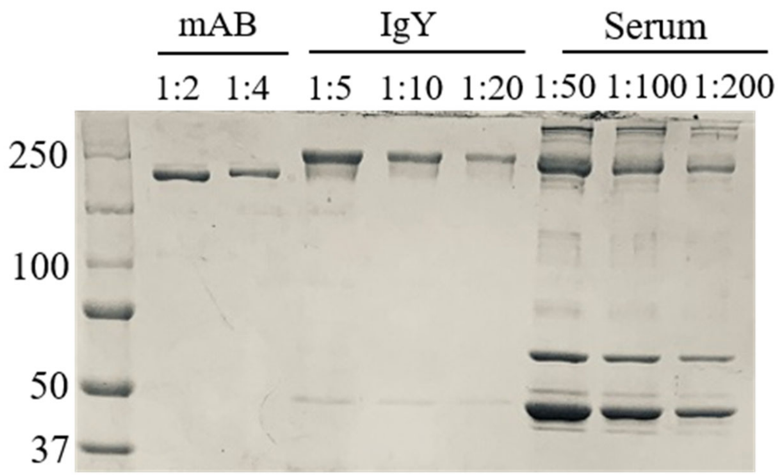


The protein concentration of mAb and egg yolk IgY were 0.34 and 2.1 mg/mL, respectively.
