# Supplementary material for: Maternal immunization against myostatin suppresses post-hatch chicken growth
Source: PLoS One. 2022 Oct 6;17(10):e0275753. doi: 10.1371/journal.pone.0275753 (PMC9536644; doi:10.1371/journal.pone.0275753)
Supplement: Attachment — Submitted filename: Response to Reviewers.docx [file pone.0275753.s005.docx]

September 13, 2022

**TO: Plos One**

We would like to thank the academic editor and reviewers for their valuable comments and suggestions on the submitted manuscript (PONE-D-22-18047). The following lists our responses to each point raised by the academic editor and reviewer.

1. We tried our best to follow PLOS ONE’s style requirements for our revised manuscript.
2. The method of sacrifice is added in the revised manuscript: “The birds were sacrificed using carbon dioxide asphyxiation at 7 weeks of age”
3. Original underlying images are provided in Supporting Information files.
4. The funder had no role in study design, data collection and analysis, decision to publish, or preparation of the manuscript.
5. As was requested, the Acknowledgements section that contained the funding information was removed in the revised manuscript.
6. The captions for Supporting Information files are added at the end of the revised manuscript:

Supporting Information

S1 Fig. DNA sequence of chMSTN optimized for E. coli expression

S2 Table. Feed composition

S3 Fig. Western blot results

S4 Fig. SDS-PAGE (7%) analysis of monoclonal anti-MSTN antibody (mAb), egg yolk IgY and serum in non-reduced condition

7. We tried our best to ensure that the reference list is complete and correct
8. The response to the reviewer’s comments is added:

(Lines 480-486)

“This study used layer chickens with broiler diets for post-hatch growth of chicks to provide enough protein needed for enhanced muscle growth induced by MSTN

suppression. Apparently, enhanced muscle growth did not happen in chicks from hens immunized against MSTN. Since layers are a slow-growing breed, it is questioned

whether the diet was a possible factor affecting the growth performance. However, as discussed previously, the growth of chicks from broiler hens immunized against MSTN was suppressed [19]. Thus, it is unlikely that the diet affected the growth performance of chicks.”

Sincerely yours,

Yong Soo Kim, Ph.D.

Department of Human Nutrition, Food and Animal Sciences

University of Hawaii
